# Integrated bioinformatics analysis reveals Netrin-1 as a key molecular link between Parkinson’s disease and heart failure

**DOI:** 10.3389/fnagi.2025.1709337

**Published:** 2025-12-29

**Authors:** Zhen Ni, Gaoge Wang, Yingyan Li, Huan Chen, Hongwei Hou, Qingyuan Hu

**Affiliations:** 1 State Key Laboratory of Membrane Biology, School of Life Sciences, Peking University, Beijing, China; 2 Beijing Life Science Academy, Beijing, China; 3 China National Tobacco Quality Supervision and Test Center, Zhengzhou, China

**Keywords:** Parkinson’s disease, heart failure, RNA-seq, Netrin-1, bioinformatics

## Abstract

**Background:**

Parkinson’s disease (PD) patients face a higher risk of developing heart failure (HF). The objective of the study was to investigate the hub genes and potential mechanisms linking Parkinson’s disease (PD) to heart failure (HF) using multiple integrative bioinformatics tools.

**Methods:**

Integrated bioinformatics analyses were performed. One HF dataset (GSE57338) and three PD datasets (GSE7621, GSE20146, GSE49036) were obtained from the GEO database. Weighted gene co-expression network analysis (WGCNA) was used to identify PD-related genes. Differentially expressed genes (DEGs) between PD and normal samples, as well as between HF and normal samples, were identified. The intersection of DEGs, WGCNA-derived PD-related genes, and genes encoding known secretory proteins was analyzed to find PD-associated secretory proteins. Immune cell infiltration in HF was assessed using CIBERSORT. Protein-protein interaction (PPI) network analysis was conducted to identify hub genes. Key findings were experimentally validated in an MPTP-induced PD mouse model through behavioral tests, ELISA, and immunohistochemistry.

**Results:**

Analysis identified 21 PD-associated secretory proteins. Intersection with HF DEGs revealed 12 common genes, from which 8 functional genes with consistent expression patterns in both conditions were identified. PPI network analysis highlighted three hub genes: *RELN, SLIT1*, and *NTN1*. Reactome pathway analysis indicated that NTN1 is involved in cardiac-related processes like muscle contraction and cardiac conduction. Experimental validation in PD model mice confirmed a significant decrease in Netrin-1 levels in the blood, striatum, and heart. Furthermore, a strong negative correlation was found between cardiac Netrin-1 expression and collagen deposition, suggesting its potential role in impacting cardiac function.

**Conclusion:**

These insights highlight the coexistence of PD and HF and suggest new avenues for investigating strategies to prevent HF in PD patients, particularly by exploring the role of Netrin-1 in the heart and its potential for cardioprotection.

## Introduction

Parkinson’s disease (PD) is a progressive neurodegenerative disorder affecting 1–3% of individuals over the age of 65, representing a significant global health challenge (Macerollo and Chen, 2016; Rocca, 2018). Emerging research indicates that PD is not only characterized by cognitive decline but also by inflammation and oxidative stress, both of which are key factors in the development of various cardiovascular diseases, including myocardial infarction (MI), ischemic stroke, and heart failure (HF) (Park et al., 2020). HF, which affects more than 60 million people worldwide with a prevalence of 2% in the adult population, is the leading cause of cardiovascular hospitalization among individuals over 60 years old (Diseases and Injuries, 2020; Roger, 2021; Tsao et al., 2022). Recent studies have indicated that PD patients face a higher risk of developing HF compared to the general population (Park et al., 2020). Cardiac alterations, including orthostatic hypotension, heart rate variability, electrocardiogram changes, and baroreflex dysfunction, can occur in both the early and late stages of PD, typically worsening as the disease progresses (Beura et al., 2024; Cuenca-Bermejo et al., 2021). Notably, approximately 80% of PD patients experience cardiovascular complications, which accelerate disease progression and significantly elevate mortality risk (Goncalves et al., 2022). HF is a leading cause of death in PD patients, with a prevalence twice that of the general population (Pennington et al., 2010). Both PD patients and MPTP-treated mouse models exhibit cardiac ultrastructural and geometric abnormalities, such as cardiac atrophy and interstitial fibrosis, alongside functional deficits including reduced E/A ratio, fractional shortening, ejection fraction, and cardiomyocyte contraction (Yu et al., 2024). These findings underscore the critical need to explore the interplay between PD and heart disease to better understand their combined impact on the heart and brain.

Recent evidence reveals a strong connection between PD and cardiac dysfunction, although with treatment options remain limited. Studies exploring the genetic and environmental factors associated with PD have identified several shared risk factors with HF, including advanced age, male gender, and diabetes mellitus (Cuenca-Bermejo et al., 2021; Potashkin et al., 2020). Additionally, common pathways such as inflammation, glucose metabolism, oxidative stress, lipid metabolism, along with shared risk factors, appear to contribute to this association (Beura et al., 2024; Korczowska-Ła̧cka et al., 2023; Potashkin et al., 2020). PD-related genes such as *PINK1*, *DJ-1*, and *LRRK2* are expressed in cardiac tissue, with evidence suggesting these proteins provide protection against cardiac damage (Billia et al., 2013; Billia et al., 2011; Dongworth et al., 2014; Zhang et al., 2018). Non-coding RNAs also play a critical role in pathways shared between PD and cardiovascular diseases, including microRNAs such as miR-124 and miR-133b, and long non-coding RNAs like MALAT1 and HOTAIR (Acharya et al., 2020). These findings suggest that PD may contribute to HF, at least partially, through secretory proteins. However, the molecular mechanisms underlying PD-related HF remain complex and are not yet fully understood.

Given the substantial prevalence and health burden of both PD and HF, understanding their shared risk factors can provide valuable insights into their underlying pathophysiological mechanisms and reveal novel targets for prevention and treatment. In this study, we employed multiple integrative bioinformatics tools to reveal the hub genes and potential mechanism underlying PD-related HF.

## Results

### Data processing

**FIGURE 1:**
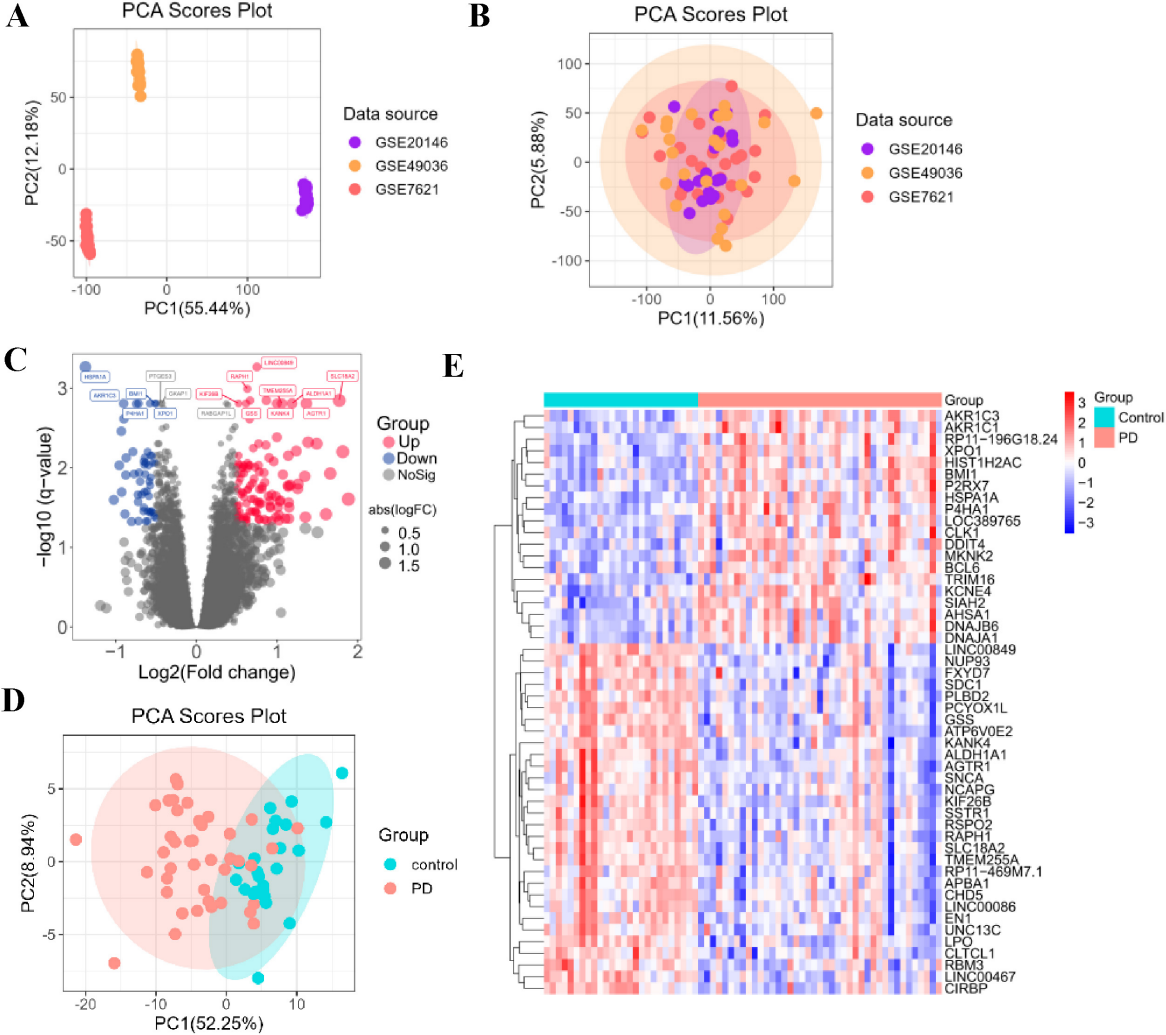
Integration of PD datasets and associated differential expression analysis. **(A)** PCA of the three original PD datasets before batch-effect correction. **(B)** PCA of the integrated PD dataset after batch-effect correction. **(C)** Volcano plot showing PD DEGs in the integrated PD dataset. Upregulated genes are presented in red dots and downregulated genes are presented in blue dots. **(D)** PCA based on the top 100 DEGs to improve separation between PD and control groups. **(E)** Heatmap showing the top-50 DEGs in the integrated PD dataset.

We obtained integrated PD expression data by applying batch correction to three datasets (GSE7621, GSE20146, and GSE49036) using the combat function from the “SVA” package. The final dataset included 27 healthy controls (HC) and 41 PD patients. Principal component analysis (PCA) of the non-integrated data showed clear batch effects across the original datasets (Figure 1A). Following batch effect correction, the samples were effectively merged, as demonstrated by the improved overlap in the PCA plot (Figure 1B).

### Identification of differentially expressed genes between PD and normal samples

Differential analysis between the combined PD and normal samples identified 168 DEGs based on an adjusted *p* ≤ 0.05 and | log_2_(fold change)| ≥ 0.5. Among these, 52 genes were upregulated, and 116 were downregulated (Supplementary Table 1). We visualized the DEGs expression patterns using a volcano plot (Figure 1C). To further enhance the separation between PD and control groups, we performed a PCA using the top 100 most significant DEGs, which revealed a clearer distinction (Figure 1D). The expression patterns of the top 50 DEGs are displayed in a heatmap, showing distinct clustering of PD and control samples and confirming the reliability of the identified transcriptional signature (Figure 1E).

### Identification of PD-related genes (PDGs) by WGCNA

**FIGURE 2:**
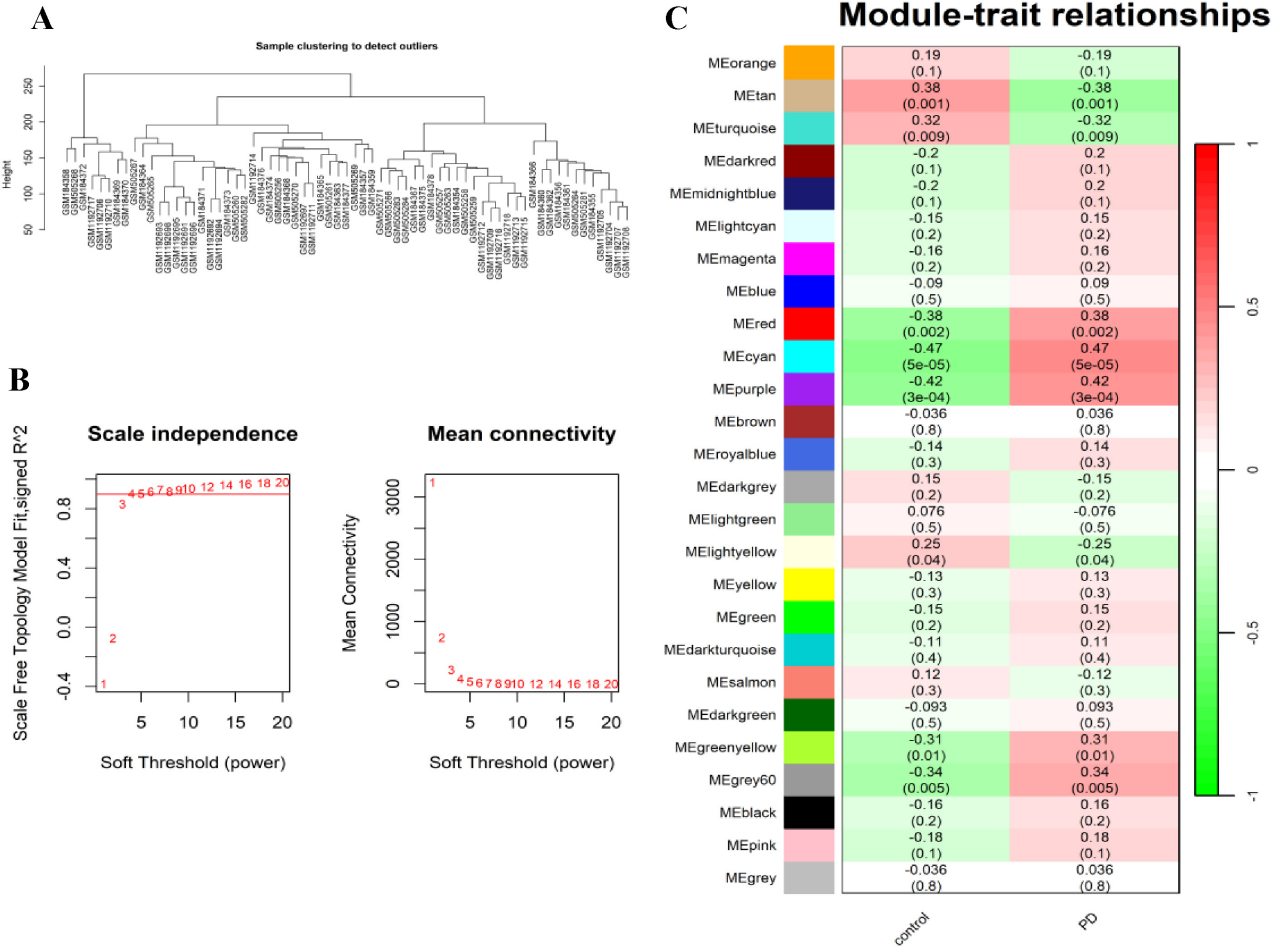
Screening of the key module genes in the integrated PD dataset via WGCNA. **(A)** Module clustering difference diagram. **(B)** Selection of soft threshold. **(C)** Correlation matrix diagram of each module. Correlation (upper) and *p*-value (bottom) of module eigengenes and PD status are presented.

To further investigate PDGs, we performed a WGCNA to identify the most relevant gene modules in PD samples. Firstly, a module clustering different graph was generated to filter out genes with minimal expression changes (Figure 2A) Using scale independence and average connectivity, we selected a soft-thresholding power of 4 (Figure 2B). This threshold generated 26 distinct gene modules, with the correlations between PD and these modules (Figure 2C). The list of genes within each module is available in Supplementary Table 2. Correlation analysis identified seven modules—cyan, tan, purple, red, turquoise, green-yellow, and grey60—as strongly associated with PD. Genes from these modules were designated as PDGs.

### Identification of differentially expressed secretory proteins in PD

**FIGURE 3:**
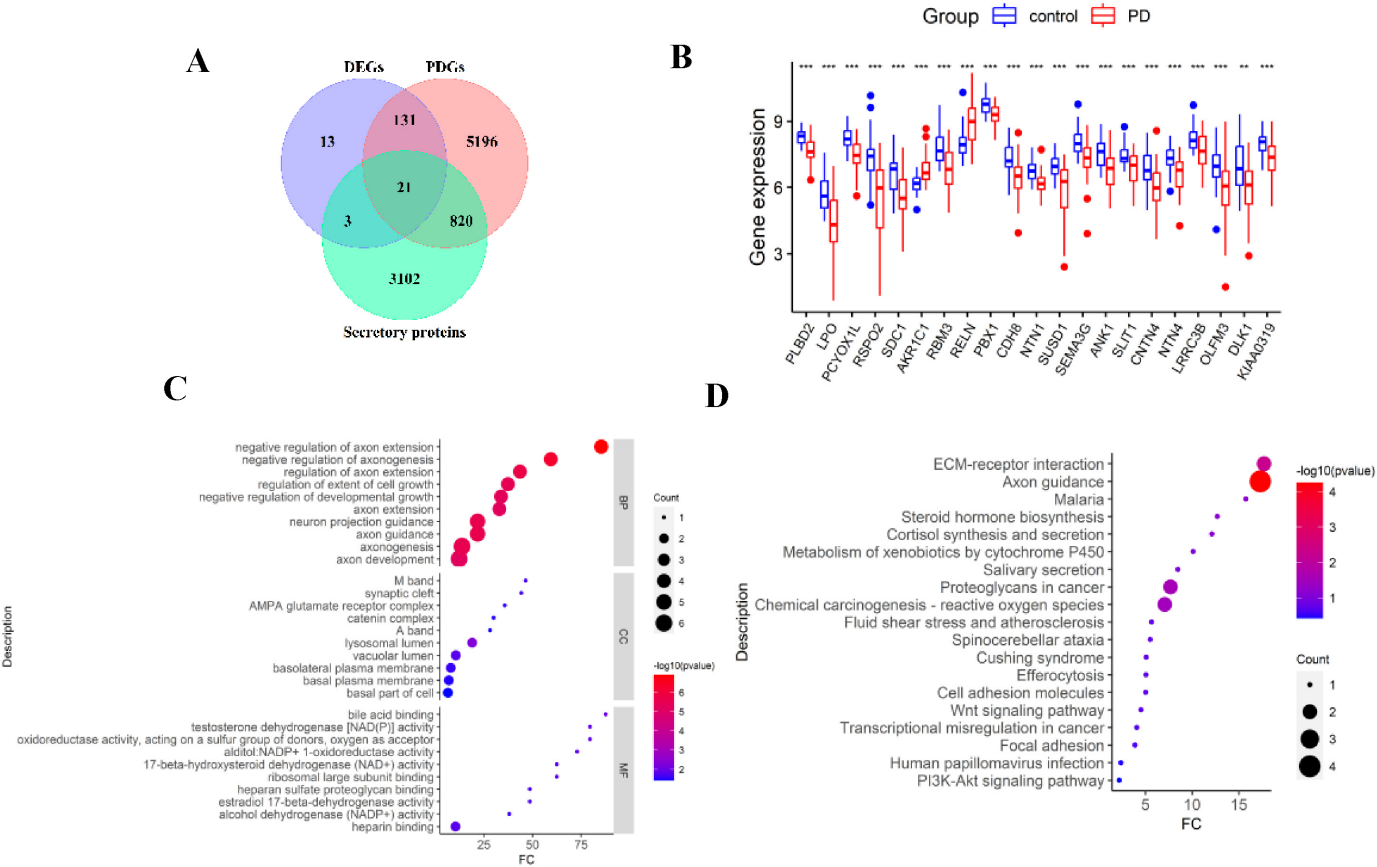
Identification of differentially expressed secretory proteins in PD. **(A)** Venn diagram showing the intersection of DEGs, PDGs, and genes coding secretory proteins. **(B)** Boxplot of the 21 genes coding secretory proteins. **(C)** GO enrichment result of the 21 genes. **(D)** KEGG enrichment result of the 21 genes.

Considering that PD may influence the onset and progression of HF by releasing secretory proteins, we identified PD-associated secretory proteins by intersecting DEGs, PDGs, and known secretory proteins. This analysis yielded 21 PD-associated secretory proteins, as depicted in the Venn diagram (Figure 3A). Comparative analysis revealed that *PLBD2*, *LPO*, *PCYOX1L*, *RSPO2*, *SDC1*, *RBM3*, *PBX1*, *CDH8*, *NTN1*, *SUSD1*, *SEMA3G*, *ANK1*, *SLIT1*, *NTN4*, *LRRC3B*, *OLFM3*, *DLK1*, and *KIAA0319* were expressed at low levels in PD samples, while *AKR1C1* and *RELN* were highly expressed (Figure 3B). We further analyzed these genes for functional enrichment using Gene Ontology (GO) and KEGG pathway analyses. The most significantly enriched GO terms were categorized into Biological Process (BP), Cellular Component (CC), and Molecular Function (MF), including processes such as negative regulation of axon extension, negative regulation of axonogenesis, and regulation of axon extension; enriched components included the synaptic cleft and basolateral plasma membrane (Figure 3C). KEGG pathway analysis identified critical pathways related to axon guidance, ECM-receptor interaction, proteoglycans in cancer, and chemical carcinogenesis involving reactive oxygen species (Figure 3D).

### Identification of differentially expressed genes in HF

**FIGURE 4:**
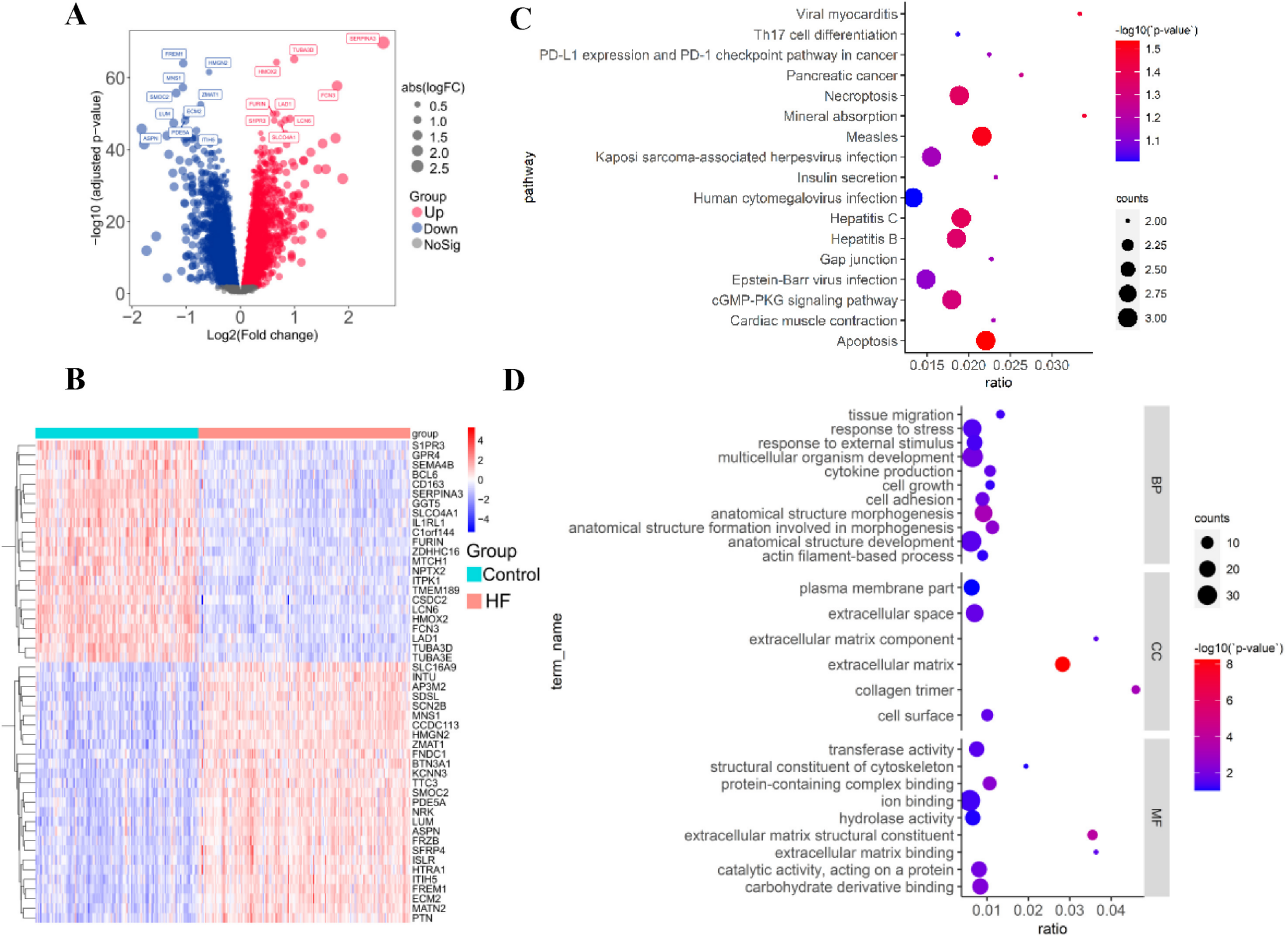
Identification of differentially expressed genes in HF. **(A)** Volcano map of DEGs between HF and normal samples in the GSE57338 dataset. **(B)** Heatmap showing the top-50 DEGs significantly upregulated or downregulated. **(C)** KEGG enrichment results of the top-100 HF DEGs. **(D)** GO enrichment result of the top-100 HF DEGs.

A total of 8,884 DEGs were identified between HF and normal samples in the GSE57338 dataset, with a cutoff value of adjusted *p* ≤ 0.01. This included 4,171 upregulated genes and 4,713 downregulated genes (Supplementary Table 3). The DEGs were visualized using volcano plots (Figure 4A) and heatmaps, with the top 50 DEGs presented in a cluster heatmap (Figure 4B). To better understand the functions and mechanisms of the pathogenic genes, functional enrichment and KEGG analysis were performed using the top 100 HF DEGs (Figure 4C). The GO analysis revealed that the most enriched BP terms were associated with anatomical structure morphogenesis (Figure 4D). For CC, the most enriched terms were related to the extracellular matrix and collagen trimer. The most enriched MF terms were linked to extracellular matrix structural constituents and protein-containing complex binding. In the KEGG analysis, the target genes were notably enriched in pathways related to cocaine addiction and calcium signaling.

### Immune cell infiltration in HF

**FIGURE 5:**
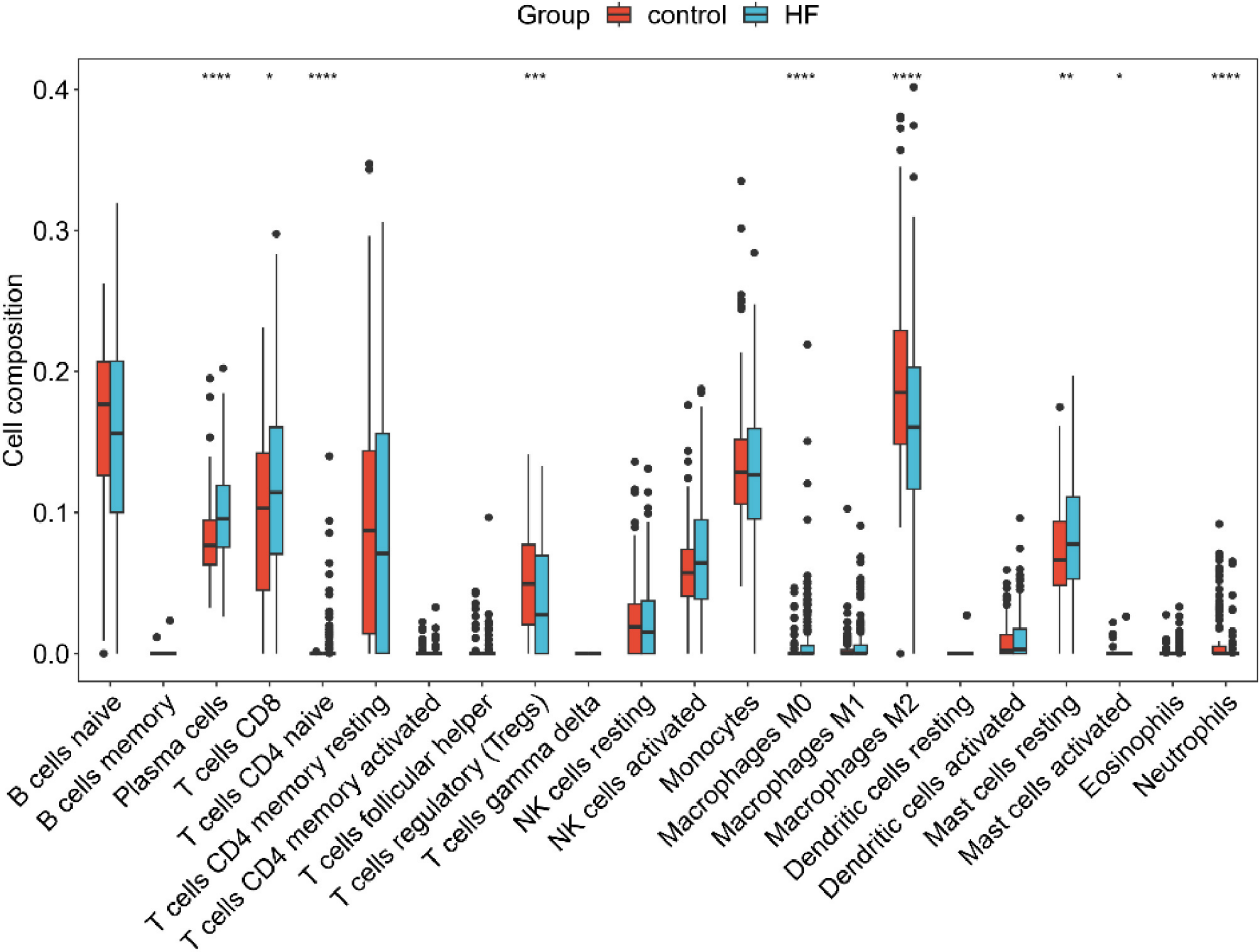
Boxplot highlighting the differences in immune cell infiltration between the normal and HF groups. *p < 0.05, **p < 0.01, ***p < 0.001, ****p < 0.0001.

CIBERSORT was employed to quantify the fractions of infiltrating immune cells in HF. The immune cell profiles for 22 immune cell types were identified in both HF and normal samples Significant differences between the normal and HF groups were observed in the myocardial infiltration of nine immune cell types (Figure 5). Specifically, plasma cells, CD8 + T cells, naive CD4 + T cells, M0 macrophages, and resting mast cells exhibited higher expression in the HF group; while Tregs, M2 macrophages, activated mast cells, and neutrophils were less abundant in the HF group.

### Identification of potential functional genes for PD-related HF

**FIGURE 6:**
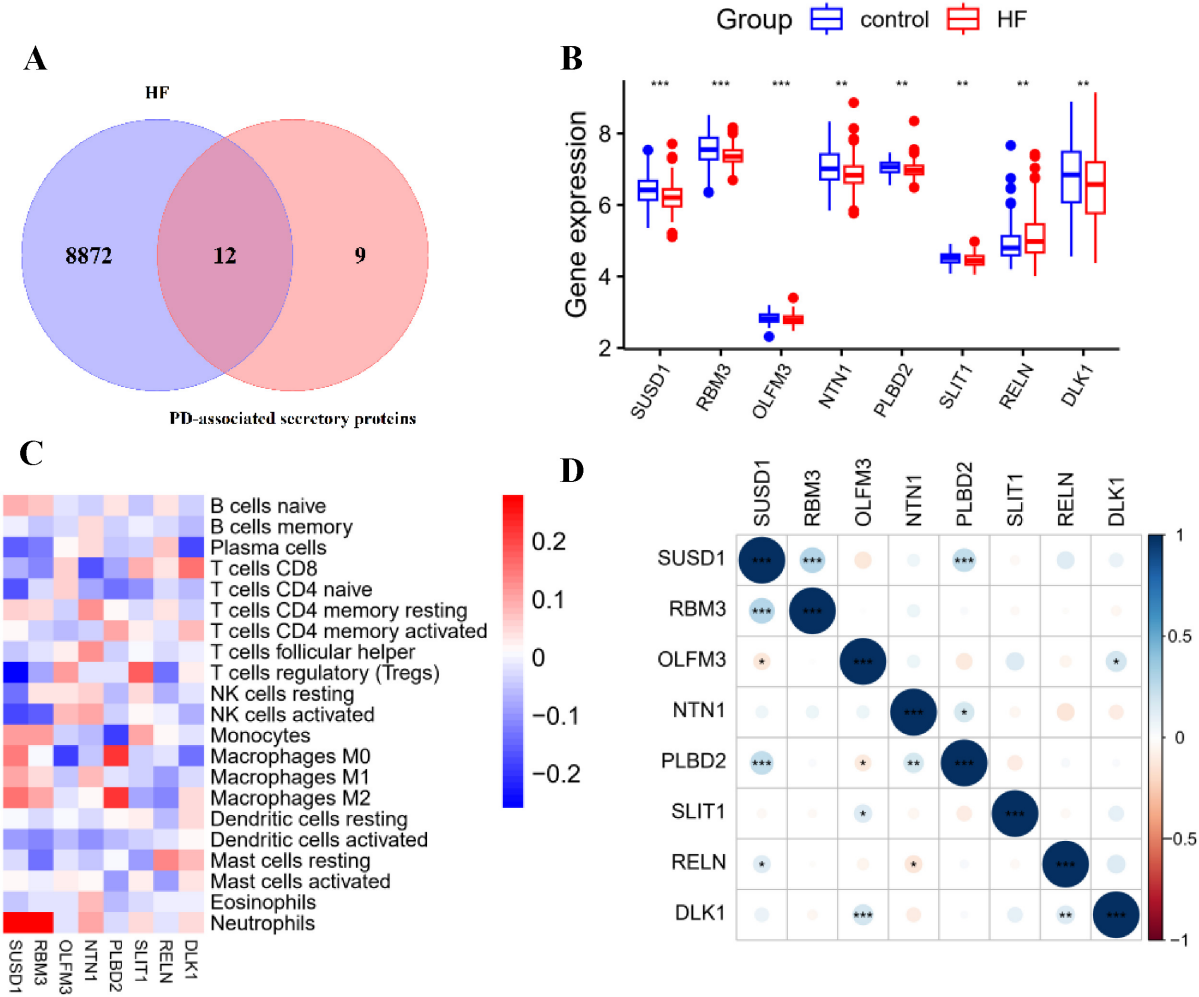
Identification and associated correlation analysis of potential functional genes for PD-related HF. **(A)** Venn diagram showing 12 overlay genes between HF DEGs and PD-associated secretory proteins. **(B)** Gene expression of the 8 functional genes in both the control and HF groups. **(C)** Heatmap indicating the correlation between immune cell infiltration scores and expression levels of the 8 key genes. **(D)** Heatmap indicating the correlation among the 8 genes by Pearson correlation analysis. *p < 0.05, **p < 0.01, ***p < 0.001.

Twelve common genes were identified at the intersection of PD-associated secretory proteins and HF DEGs, suggesting their potential critical roles in HF related to PD (Figure 6A). We identified 8 key functional genes exhibiting consistent expression patterns in both PD and HF. Specifically, *RELN* was more highly expressed in control samples, whereas the remaining genes showed higher expression in HF samples (Figure 6B). Further analysis revealed correlations between the expression of these 8 genes and the proportion of differentially infiltrated immune cell types. *SUSD1* and *RBM3* were significantly correlated with neutrophils in HF (Figure 6C). Additionally, *SUSD1*, *OLFM3*, *PLBD2*, and *DLK1* were significantly correlated with M0 macrophages in HF (Figure 6C). Correlation analysis of the 8 potential functional genes revealed that *NTN1* exhibited a significantly positive correlation with *RELN* (Figure 6D).

### Identification of key genes by PPI network analysis

**FIGURE 7:**
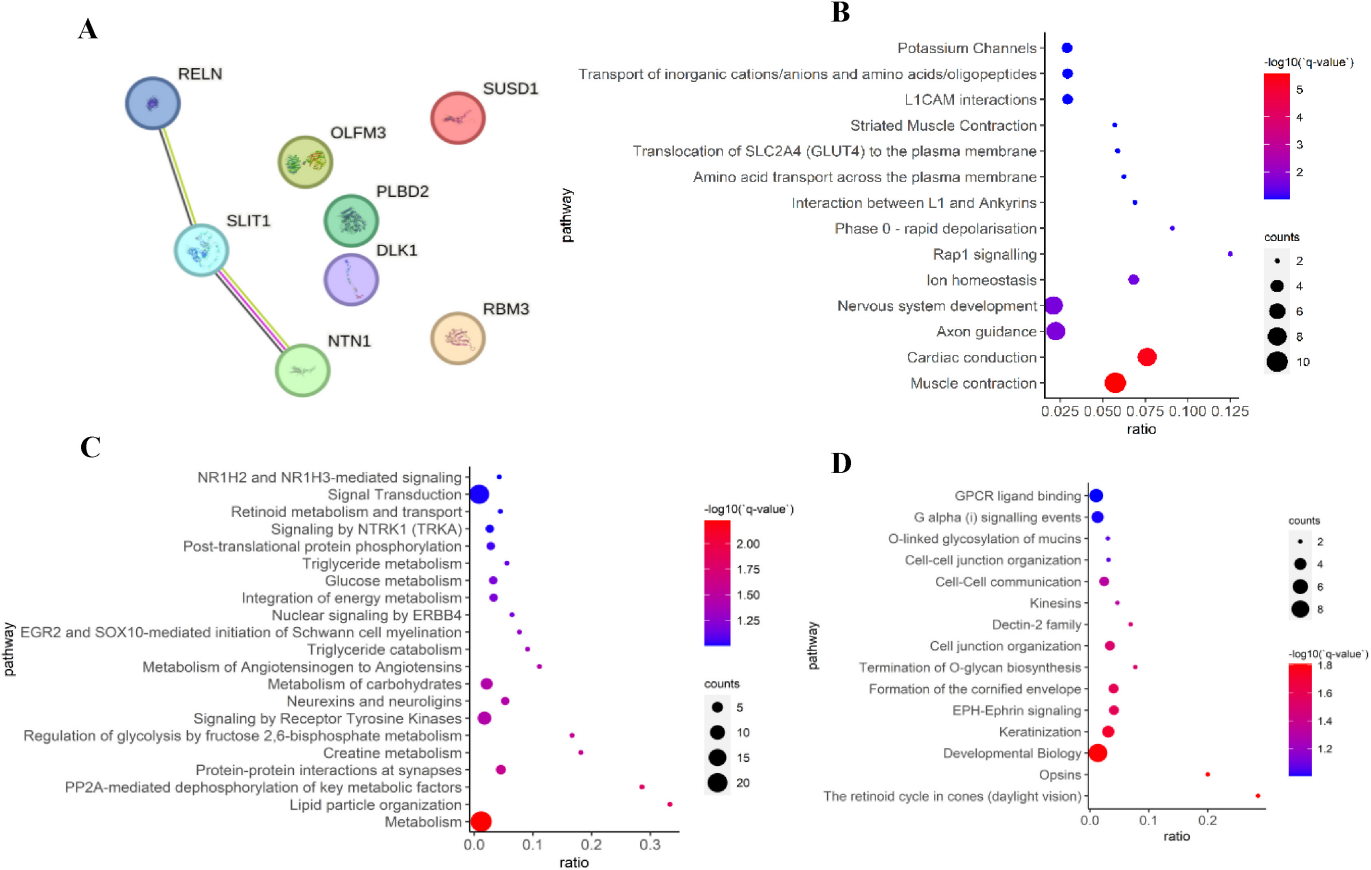
Identification of key genes via PPI network analysis and pathway enrichment analysis. **(A)** Three key genes identified via a PPI network analysis of the 8 overlayed genes. **(B)** REACTOME enrichment result of the top 100 genes significantly correlated with *NTN1* in the GSE57338 dataset. **(C)** REACTOME enrichment result of the top 100 genes significantly correlated with *RELN* in the GSE57338 dataset. **(D)** REACTOME enrichment result of the top 100 genes significantly correlated with *SLIT1* in the GSE57338 dataset.

To identify hub genes among the 8 potential functional genes, a PPI network was constructed using the STRING database and analyzed with Cytoscape. This analysis revealed a sub-network consisting of three candidate genes: *RELN*, *SLIT1*, and *NTN1* (Figure 7A). To further explore the potential roles of these genes in HF, we conducted REACTOME enrichment analysis on the top 100 genes that showed significant correlations with *RELN*, *SLIT1*, and *NTN1* in the GSE57338 dataset (Figures 7B–D). The REACTOME analysis indicated that *NTN1* may be involved in processes such as muscle contraction, cardiac conduction, and striated muscle contraction, with enriched genes including key regulators of cardiac excitability and calcium handling like *TRDN, STIM1, KCNQ1, SCN5A*, and *KCNIP2* (Bénard et al., 2016; Hesse et al., 2007; Stein et al., 2011). This strong association with core cardiac functions, coupled with literature evidence that Netrin-1 modulates cardioprotective pathways against ischemia-reperfusion injury and hypertrophy (Bénard et al., 2016), led us to select *NTN1* for further experimental validation.

### Experimental validations of Netrin-1 expression in PD mice

**FIGURE 8:**
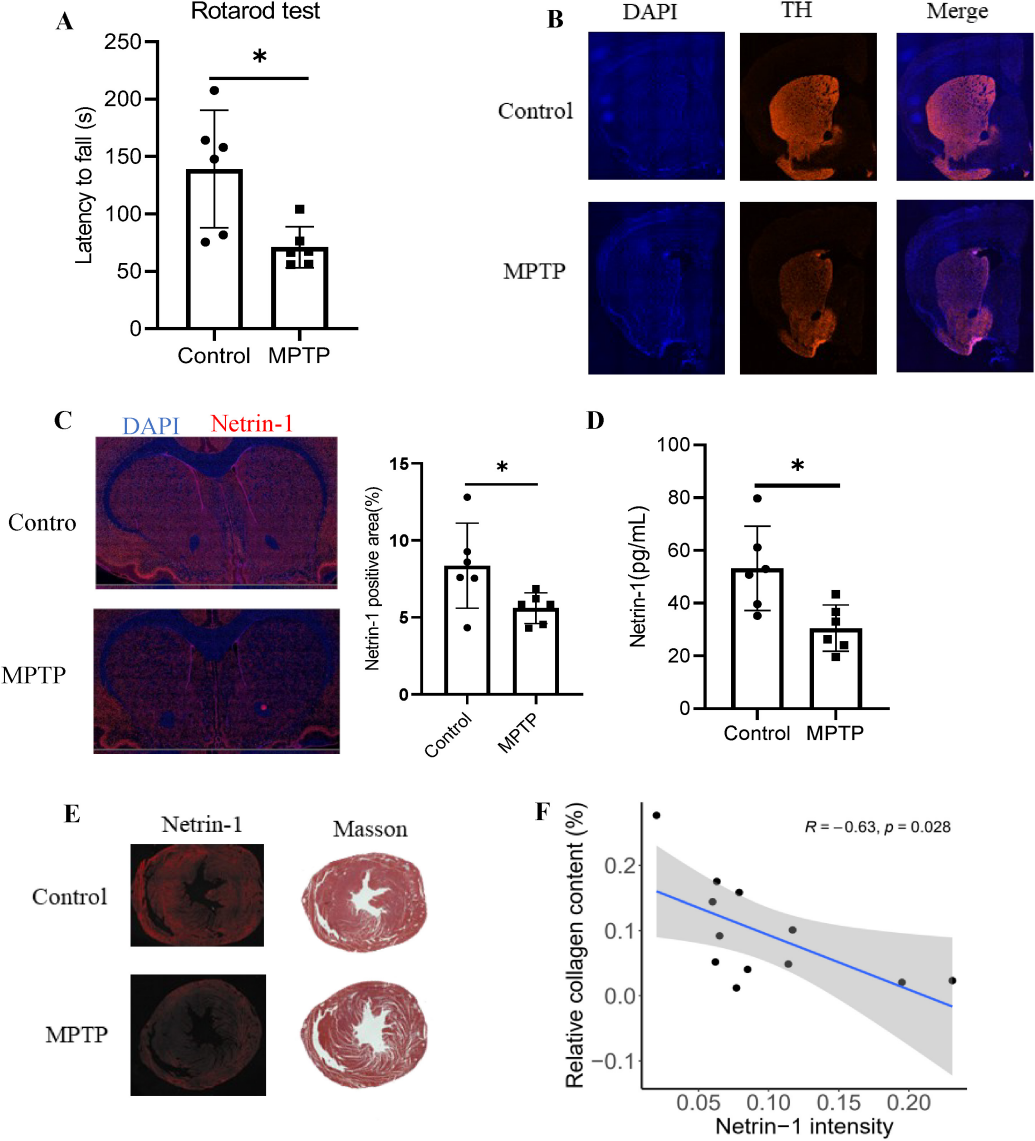
Netrin-1 decreased in both the brain and heart of PD mice. **(A)** Rotarod test evaluating the motor coordination of mice. **(B)** Immunofluorescent studies of TH in the striatum. **(C)** Immunofluorescent studies of Netrin-1 in the striatum. **(D)** ELISA analysis demonstrating the plasma concentration of Netrin-1. **(E)** Immunofluorescent studies of Netrin-1 and Masson’s trichrome staining in the heart. **(F)** Correlation between Netrin-1 intensity and relative collagen content in the heart. Data represent mean ± SEM (*n* = 6 per group). *p < 0.05.

To investigate the role of Netrin-1 as a potential link between PD and HF, we established a PD mouse model by administering MPTP (20 mg/kg) twice weekly for 4 weeks. MPTP treatment induced significant motor impairment, as evidenced by a shorter latency to fall in the rotarod test (Figure 8A). Consistent with PD pathology, we observed a substantial loss of dopaminergic neurons in the striatum, indicated by reduced tyrosine hydroxylase (TH) staining intensity (Figure 8B). Crucially, and providing a molecular link to cardiac dysfunction, Netrin-1 expression was significantly decreased not only in the striatum (Figure 8C) but also in the plasma (Figure 8D) and the heart of MPTP-treated mice (Figure 8E). To directly assess MPTP-induced cardiotoxicity and its relation to Netrin-1, we evaluated cardiac fibrosis using Masson’s trichrome staining, which quantifies collagen accumulationumuwell-established indicator of impaired cardiac function. Our analysis revealed a strong negative correlation between cardiac Netrin-1 levels and the extent of fibrosis (Figures 8E,F). These results demonstrate that MPTP modeling recapitulates key features of both PD and HF, and they position Netrin-1 as a critical molecule whose depletion in the heart is associated with the progression of fibrosis, thereby contributing to the pathological interplay between the brain and the heart in PD.

## Discussion

The objective of this study was to identify key hub genes in PD patients that may play a functional role in HF. To detect PD-associated secretory proteins, we performed differential expression analysis and WGCNA, identifying 21 genes through the intersection of DEGs, PDGs, and secretory proteins. We further identified 8 functional genes by intersecting these 21 PD-associated secretory proteins with HF DEGs. Using the CIBERSORT algorithm, we explored immune cell infiltration in HF. The PPI network analysis highlighted *RELN*, *SLIT1*, and *NTN1* as shared hub genes between PD and HF, making them as the most relevant to both conditions. Reactome pathway analysis suggested that *NTN1* may be involved in processes such as muscle contraction, cardiac conduction, and striated muscle contraction. Based on these findings, we hypothesized that *NTN1* plays a pivotal role in the coexistence of PD and HF. To investigate this further, Experimental validation in MPTP-induced PD models confirmed significant reduction of Netrin-1 protein levels in plasma, while immunohistochemical analysis demonstrated concurrent depletion in both striatal and cardiac tissues. Most importantly, we established a strong negative correlation between cardiac Netrin-1 expression levels and collagen deposition, indicating a direct association between Netrin-1 depletion and the progression of myocardial fibrosis. These findings collectively position Netrin-1 as a key molecular mediator in the pathological interplay between neurodegenerative and cardiovascular systems in PD.

While our bioinformatic and experimental analyses identified *RELN, SLIT1*, and *NTN1* as independent hub genes, emerging evidence suggests potential functional crosstalk among these molecules. Notably, all three genes encode secreted proteins that belong to the axon guidance pathway, which plays crucial roles in neural development and maintenance. Specifically, both Netrin-1 (encoded by *NTN1*) and Slit1 can interact with their respective receptors (DCC/UNCS and Robo receptors) to modulate cytoskeletal reorganization, while Reelin (encoded by *RELN*) signals through ApoER2/VLDLR receptors to regulate cell adhesion and positioning, indicating convergent downstream signaling pathways (Botella-López et al., 2006; Calvier et al., 2021; Jiang et al., 2019). Furthermore, these guidance molecules have been implicated in modulating inflammatory responses: Netrin-1 suppresses leukocyte migration (Rosenberger et al., 2009; Tadagavadi et al., 2010), Slit-Robo signaling regulates macrophage chemotaxis (Geraldo et al., 2021; Mommersteeg et al., 2015; Zhao and Mommersteeg, 2018), and Reelin influences vascular inflammation (Alexander et al., 2023; Krueger et al., 2020), suggesting their collective involvement in the neuro-inflammatory and cardio-inflammatory axes linking PD and HF. Although direct interactions among these three proteins remain unexplored, their co-expression in neural and cardiac tissues and overlapping roles in cellular guidance mechanisms posit a synergistic network worthy of future investigation.

Reelin, a 420-kDa extracellular glycoprotein, plays essential roles in development, circuit maturation, and synapse maintenance in the central nervous system (CNS) (Frotscher, 2010; Yu et al., 2015). It binds to the transmembrane receptors apolipoprotein receptor 2 and very-low-density lipoprotein receptor, which transduce the Reelin signaling through the intracellular adapter disabled-1 (Botella-López et al., 2006). Studies have shown that Reelin is upregulated in the brain and cerebrospinal fluid (CSF) in several neurodegenerative diseases, including PD (Botella-López et al., 2006). Beyond the CNS, Reelin is expressed in peripheral tissues such as the heart and is detectable in the blood (Smalheiser et al., 2000). Recent research has implicated the pharmacological depletion of circulating Reelin inhibits the atherosclerosis progression (Calvier et al., 2021). Reelin is also expressed in cardiac Schwann cells and lymphatic endothelial cells. It promotes neonatal cardiomyocyte proliferation and is required for efficient heart repair and function after neonatal MI (Liu et al., 2020; Pei et al., 2023). Taken together, these studies highlight the vital roles of Reelin in both the brain and heart. Further research is needed to elucidate the complex mechanisms by which Reelin contributes to the pathogenesis of HF and PD.

The Slit family (Slit1, Slit2, and Slit3) consists of secreted proteins that mediate positional interactions between cells and their environment during development by signaling through ROBO receptors (Robo1, Robo1, Robo1, and Robo4). The Slit-Robo signaling pathway plays a crucial role in axonal repulsion, axon guidance, neuronal migration, and the formation of the vascular system (Jiang et al., 2019). Migrating neurons actively regulate the formation and maintenance of their migration route by secreting the diffusible protein Slit1, which binds to its receptor, Robo, expressed on astrocytes. The Slit-Robo pathway is essential for the morphological and organizational changes in astrocytes that lead to the formation and maintenance of astrocytic tunnels (Kaneko et al., 2010). In an experimental stroke model, neuroblasts overexpressing Slit1 were transplanted into the post-stroke brain. These neuroblasts migrated closer to the lesion site than control neuroblasts, matured into striatal neurons, efficiently regenerated neuronal circuits, and promoted functional recovery in the mice (Kaneko et al., 2018). Beyond the CNS, accumulating evidence from various animal models indicates that the Slit-Robo signaling pathway plays a critical role in multiple aspects of heart development, including cardiac cell migration and alignment, lumen formation, chamber morphogenesis, and the formation of the ventricular septum, semilunar and atrioventricular valves, caval veins, and pericardium (Mommersteeg et al., 2015; Zhao and Mommersteeg, 2018). Despite these findings, the role of Slit-Robo signaling in the pathogenesis of HF and PD remains underexplored.

Netrin-1 is predominantly expressed in the brainstem, particularly in the substantia nigra. Studies have shown a significant decrease in Netrin-1 levels in both PD patients and animal models of PD, with plasma Netrin-1 levels significantly reduced in PD patients. This reduction correlates with the severity of PD symptoms (Ahn et al., 2021; Hua et al., 2023). Silencing endogenous Netrin-1 in the substantia nigra of adult mice resulted in a dramatic 70% loss of dopaminergic neurons. In contrast, administration of recombinant Netrin-1 protein in the MPTP/SNCA mouse model and in 6-OHDA-lesioned rats led to significant neuroprotective and neurorestorative effects on mature nigral dopamine neurons (Jasmin et al., 2021). Aerobic exercise has been shown to increase serum and myocardial Netrin-1 levels and upregulate the DCC receptor while decreasing the expression of myocardial MMP2 and MMP9 proteins. This modulation improves the degree of fibrosis following MI in rats (Bader et al., 2019). Clinically, patients with impaired myocardial perfusion often present with ischemic heart disease symptoms such as exertional angina and shortness of breath. Atherosclerotic plaque rupture can result in acute coronary artery occlusion, leading to MI. Survivors of an MI may eventually develop HF, depending on the extent of the infarcted myocardial area.

Netrin-1 has demonstrated cardioprotective effects in ischemia-reperfusion injury (IRI) following MI (Layne et al., 2015). *In vitro* studies suggest that Netrin-1’s cardioprotective actions in IRI are primarily mediated by a DCC-dependent increase in nitric oxide (NO) production from both endothelial cells and cardiomyocytes (Nguyen and Cai, 2006; Siu et al., 2015; Zhang and Cai, 2010). Netrin-1 promotes cardioprotection by inhibiting NOX4 activity, which facilitates the recoupling of nitric oxide synthase (NOS), enhances NO bioavailability, reduces oxidative stress, and ultimately, preserves mitochondrial function (Li et al., 2015; Siu et al., 2015). Additionally, Netrin-1 inhibits leukocyte migration and reduces inflammation-mediated tissue injury (Rosenberger et al., 2009; Tadagavadi et al., 2010). Netrin-1 has been shown to prevent MI caused by ischemia-reperfusion and to suppress the development of cardiac hypertrophy and heart failure induced by transverse aortic constriction (Durrani et al., 2012; Zhang and Cai, 2010). Furthermore, PGE1 has been found to mitigate angiotensin II (AngII)-induced cardiac hypertrophy by activating of the EP3 receptor, which upregulates Netrin-1 and inhibits the downstream MAPK signaling pathway (Shen et al., 2021).

Together, these findings strongly suggest that small Netrin-1-derived peptides are highly effective in protecting the heart from myocardial ischemia-reperfusion injury, highlighting their potential as peptide-based therapeutics for treating MI (Li and Cai, 2015). Collectively, the studies emphasize the critical role of Netrin-1 in both the brain and the heart. However, further investigation is necessary to fully understand the complex mechanism by which Netrin-1 contributes to the pathogenesis of heart failure and Parkinson’s disease.

## Conclusion

In conclusion, we identified three pivotal genes—*RELN*, *SLIT1*, and *NTN1*—that bridge the connection between PD and HF. Our findings demonstrate that Netrin-1 levels in the blood are altered in PD models, potentially impacting heart function. This study provides a foundation for future research into the molecular mechanisms underlying the interplay between PD and HF. These insights highlight the coexistence of PD and HF and suggest new avenues for investigating strategies to prevent HF in PD patients, particularly by exploring the role of Netrin-1 in the heart and its potential for cardioprotection.

## Materials and methods

### Data acquisition and processing

We downloaded three PD datasets (GSE7621, GSE20146, and GSE49036) from the GEO database,^1^ merged them, and used the combined data as the training set. Additionanlly, we obtained the microarray dataset for heart tissues (GSE57338) from HF patients from GEO. The GSE57338 dataset included 313 cardiac muscle samples from 177 HF patients and 136 healthy controls All PD datasets (GSE7621, GSE20146, GSE49036) were profiled on the GPL570 platform. To integrate the PD data, we performed batch correction on the three PD datasets using the “combat” function from the “SVA” package in R software (version 4.2.1). This resulting integrated dataset comprised 27 HC and 41 PD patients. PCA was used to confirm the effective removal of inter-batch differences.

### DEGs analysis

We performed background correction, normalization, and gene symbol conversion on both the integrated PD dataset and the HF dataset. DEGs in the PD and HF datasets were identified using the “Limma” package in R software (Ritchie et al., 2015). For the PD dataset, DEGs were selected based on the thresholds of adjusted *p* ≤ 0.05 and | log_2_(fold change)| ≥ 0.5. In contrast, DEGs in the HF dataset were identified using the threshold of adjusted *p* ≤ 0.01. We visualized the expression patterns of DEGs using volcano plots and heatmaps, generated with the “ggplot2” and “pheatmap” packages in R software, respectively.

### WGCNA and key module genes identification

We utilized the R package “WGCNA” to identify gene modules with correlated expression patterns and to explore the relationship between gene networks and phenotypes (Langfelder and Horvath, 2008). The optimal soft threshold was determined using the pickSoftThreshold function, with a threshold of 4 identified as the best fit. Based on this threshold, we constructed a scale-free network, followed by the creation of a topological matrix and hierarchical clustering. We calculated module eigengenes by dynamically cutting the identified gene modules, ensuring that each module contained at least 50 genes. Inter-module correlations were constructed based on module eigengenes. The correlation between modules and clinical features was determined through Spearman correlation analysis. Within each identified module, genes with the highest connectivity (hub genes) were identified as central and considered potential key drivers of module function for further analysis.

### Secretory proteins access

Secretory proteins were obtained from The Human Protein Atlas database^2^ (Uhlen et al., 2010). A total of 3,970 genes encoding secretory proteins were downloaded from the protein class “SPOCTOPUS predicted secreted proteins”.^3^

### The construction of PPI network

To investigate interactions between PD-associated secretory proteins and key HF-related genes, we constructed a PPI network based on the STRING database^4^ with a medium confidence score threshold of > 0.4. The PPI network was then visualized using Cytoscape software (version 3.8.2).

### Immune infiltration analysis

We employed the “CIBERSORT” package to evaluate immune cell infiltration from the HF gene expression profile (Chen et al., 2018). First, we uploaded the gene expression matrix data into CIBERSORT and combined it with the LM22 eigengene matrix to filter samples based on a significance threshold of *p* < 0.05. This approach allowed the generation of the immune cell infiltration matrix. We then utilized the R programming package ggplot2 to create bar graphs that display the distribution of 22 immune cell types across the samples. Next, we assessed the relationships between these 22 immune cell types using the “corrplot” package. For correlation analysis between the expression of diagnostic biomarkers and the content of infiltrated immune cells, we applied Spearman’s rank correlation coefficient, with *p* < 0.05 considered statistically significant.

### Enrichment analysis of DEGs

We conducted enrichment analysis of DEGs to explore the biological functions and pathways of the candidate hub genes. Functional enrichment analyses were performed using GO, Reactome, and KEGG pathways with the clusterProfiler R package (version 4.1.3). A significance threshold of *p* < 0.05 was applied to identify meaningful associations between the hub genes and relevant pathways.

### Animal preparation

We used sixteen-month-old male C57BL/6J mice obtained from Beijing Vital River Laboratory Animal Technology Co., Ltd (Beijing, China) for this study. The mice were housed under a 12-h light–dark cycle with unrestricted access to food and water. MPTP (20 mg/kg) was administered twice a week for 4 weeks. Mice in the control group received equivalent volumes of distilled water and 0.9% saline.

### Behavioral tests

At the end of the treatment period, all mice underwent 3 consecutive days of behavioral training prior to testing. The rotarod test was used to assess motor coordination in the PD mice, employing an accelerating rotarod apparatus. The rotational speed was consistently set to 40 revolutions per minute (rpm) to standardize the test. Each experiment was conducted three times.

### Histological analysis

Heart tissues were fixed in 4% formalin and embedded in paraffin. Collagen deposition was detected by Masson’s trichrome staining, with collagen area percentage analyzed using ImageJ software.

### Enzyme-linked immunosorbent assay

The levels of Netrin-1 from mice plasma were determined using ELISA kits (Shanghai Jianglai, JL44728), read at an absorbance of 450 nm, and expressed as pg/mL.

### Immunohistochemistry staining

We fixed mouse brain tissues in 4% paraformaldehyde/30% sucrose in PBS, followed by permeabilization with PBS-T [50 mM Tris⋅HCl, 150 mM NaCl, 3% bovine serum albumin (BSA), 0.1% Triton-X100, pH 7.4]. For pathological analysis of PD, the sections or slides from the fixed and sliced samples were incubated with primary antibodies against TH (Merck, Cat#AB152) and Netrin-1 (Proteintech, Cat#20235-1-AP). Following this, the sections or slides were incubated with a fluoroconjugated secondary antibody for 1 h at room temperature. After washing three times with PBS, we stained the slides with DAPI for 5 min, followed by three additional PBS washes. Finally, we mounted the slides with a glass coverslip using mounting solution.

### Statistical analysis

We performed all data calculations and statistical analyses using R programming (version 4.1.2). For comparisons between two groups of continuous variables, we assessed normally distributed data using independent Student’s *t*-tests, while non-normally distributed data were analyzed with the Mann–Whitney U test (Wilcoxon rank sum test). We conducted correlation analysis using the Pearson correlation test for the two datasets. All statistical tests were two-sided, and a *p* < 0.05 was considered statistically significant.

## Data Availability

The datasets presented in this study can be found in online repositories. The names of the repository/repositories and accession number(s) can be found in this article/Supplementary material.

## Footnotes

1
https://www.ncbi.nlm.nih.gov/geo/

2
https://www.proteinatlas.org/

3
https://www.proteinatlas.org/search/protein_class%3ASPOCTOPUS+predicted+secreted+proteins

4
https://cn.string-db.org/
